# With a little help from my friends: adopting a P-E fit perspective in understanding the value of organizational learning climate for sustainable employability

**DOI:** 10.3389/fpsyg.2023.1128535

**Published:** 2023-04-17

**Authors:** Tinka Van Vuuren, Beatrice I. J. M. Van der Heijden, Judith H. Semeijn

**Affiliations:** ^1^Loyalis Kennis & Consult, Heerlen, Netherlands; ^2^Institute for Management Research, Radboud University, Nijmegen, Netherlands; ^3^Faculty of Management, Open Universiteit, Heerlen, Netherlands; ^4^Department of Marketing, Innovation and Organisation, Ghent University, Ghent, Belgium; ^5^School of Business, Hubei University, Wuhan, China; ^6^Kingston Business School, Kingston University, London, United Kingdom; ^7^Research Centre for Education and the Labour Market (ROA), Maastricht University, Maastricht, Netherlands

**Keywords:** sustainable employability, work ability, organizational learning climate, career commitment, age, self-perceived employability, vitality

## Abstract

**Purpose:**

The objective of our study was to investigate how organizational learning climate (measured as developmental opportunities and team support for learning), career commitment, and age are related to employees’ self-perceived employability, vitality and work ability (e.g., their sustainable employability). Our study adopted a P-E fit perspective building upon the notion that sustainable employability is a function of both the person (P) and the environment (E) and tests a three-way interaction between organizational learning climate, career commitment, and age.

**Design:**

In total, 211 members of the support staff of a Dutch university completed a survey. Hierarchical stepwise regression analysis was used to analyze the data.

**Findings:**

Only one of the two dimensions of organizational learning climate that we measured, namely the developmental opportunities, appeared to be associated with all indicators of sustainable employability. Career commitment only had a direct positive relationship with vitality. Age was negatively related to self-perceived employability and to work ability, but not to vitality. The relationship between developmental opportunities and vitality was negatively influenced by career commitment (a negative two-way interaction effect), while a positive three-way interaction effect was found between career commitment, age, and development opportunities, and with self-perceived employability as the outcome.

**Theoretical and practical implications:**

Our findings confirmed the relevance of adopting a P-E fit perspective on sustainable employability, and of considering the possible role of age in this. It requires more detailed analyses in future research to unravel the role of age in the shared responsibility for sustainable employability. In practice, the results of our study imply that organizations should provide all employees with a working context that facilitates learning, however, with a special focus on older employees, for whom it is a particular challenge to protect their sustainable employability, possibly due to age-related stereotyping.

**Originality:**

Our study adopted a P-E fit perspective on sustainable employability and examined the association between organizational learning climate and all three components of sustainable employability: self-perceived employability, vitality and work ability. Moreover, it investigated whether and how the employee’s career commitment and age influence this relationship.

## 1. Introduction

In many Western societies, the retirement age has been raised in recent years, making it unaffordable for many employees to leave their job prematurely. In line with that, the pressure on individuals to participate in society, by means of carrying out paid work for as long as possible, is increasing (Oude Mulders et al., 2018). Therefore, it is necessary to maintain individuals’ sustainable employability across the entire career span. This requires efforts and a shared responsibility for sustainable employability from both employer and employee (Clarke and Patrickson, 2008; Veld et al., 2015). Our scholarly work is based on the person-environment (P-E) fit perspective (Kristof, 1996; Schneider, 2001; Kristof-Brown and Billsberry, 2013). According to this P-E perspective, human behavioral outcomes are a function of both the person (P) and the environment (E) (Lewin, 1951). Person-Environment (P-E) fit theory is a broadly used guiding framework for scholars which can help us to understand employees’ emotions, attitudes and behaviors in the workplace. In this particular study, we focus on sustainable employability as an outcome of the fit between employee (Person) and employer (Environment).

To keep up with the latest requirements of the labor market, especially in terms of lifelong learning and development of employees, the employer is responsible for creating a positive organizational learning climate. Learning climate comprises *the perceptions of work settings that may help or hinder learning at work* (Nixon, 1991), and we posit that the contribution of employers in terms of shaping positive perceptions about this learning climate is crucial, yet as a function of the fit with characteristics of the employee (i.e., individual agency), for example as expressed in their career commitment.

Career commitment refers to “*one’s attitude toward one’s profession or vocation*” (Blau, 1985, p. 278) or to*: “the identification with and active involvement in one’s own career progression*” (Van der Heijden et al., 2022, p. 566). It reflects one’s motivation to work in the chosen vocation (Carson and Bedeian, 1994), with individual agency being its core. Career commitment is already known to affect employability (Van der Heijden et al., 2022). We argue that this agency concept, together with a positive learning climate in the organization, might strengthen its impact on sustainable employability. In earlier scholarly work, the learning climate has already been studied as a moderator in the relationships between team building and employee empowerment on the one hand and employee competencies on the other (Potnuru et al., 2018). However, to the best of our knowledge, the interaction effect between learning climate and career commitment on sustainable employability is not explored yet in empirical work.

In addition to career commitment, we contend that age is a relevant factor for the individual’s functioning on the labor market: the older the employees, the more problematic their (sustainable) employability (De Lange et al., 2021). Therefore, it is important to understand how age interacts with the organizational learning climate as well as one’s own career commitment in light of sustainable employability outcomes.

To date, there is a serious lack of research investigating three-way interactions between relevant factors concerning both employer and employee responsibilities. In order to close this gap, the current scholarly work investigated the role of organizational learning climate and the employee’s career commitment, on the one hand, and sustainable employability outcomes, on the other hand. In addition, the role of age was studied, as age plays a crucial role for sustainable employability over the career span, not only because of the aging process itself, but also because of age stereotypes (Truxillo et al., 2015). We therefore included age as a third-way interaction variable in order to understand its impact in our research model.

### 1.1. Sustainable employability from a P-E fit perspective

The P-E fit perspective for sustainable employability is represented in the following definition by Van der Klink et al. (2016): “*Sustainable employability means that, throughout their working lives, workers can achieve tangible opportunities in the form of a set of capabilities. They also enjoy the necessary conditions that allow them to make a valuable contribution through their work, now and in the future, while safeguarding their health and welfare. This requires, on the one hand, a work context that facilitates this for them and on the other, the attitude and motivation to exploit these opportunities”* (Van der Klink et al., 2016, p. 47). This definition, which is based on the capability construct of Sen (2009), proposes that in order to enhance employees’ sustainable employability, two conditions need to be met: (1) organizations should facilitate an enabling working context, and (2) employees should have the attitude and motivation to seize the opportunities that arise from an enabling working context. According to Fleuren et al. (2016), the merit of this definition is that thorough attention to sustainable employability of employees is described as a shared responsibility of employer and employee, and that work and individual contextual factors together influence the employee’s sustainable employability (De Lange et al., 2013; Van der Heijden et al., 2015; Veld et al., 2015; Fleuren et al., 2016).

### 1.2. Aspects of sustainable employability

Despite the value of the definition by Fleuren et al. (2016) and Van der Klink et al. (2016) pointed out that it does not indicate how sustainable employability should be measured. Moreover, they argued that despite the fact that the employee’s work context may be related to their ability to be sustainably employed, it cannot be conceived as being part of the individual’s sustainable employability, as sustainable employability is an individual-level construct. Therefore, this article adopts the individual-level operationalization of sustainable employability as launched by Semeijn et al. (2015). They conceptualized sustainable employability as the degree to which individuals are able and willing to perform their current and future work, and operationalized the concept into a construct comprising of three components: self-perceived employability, vitality, and work ability (Van Vuuren, 2012; Semeijn et al., 2015).

Employability refers to the ability to continue to accomplish different tasks and occupations in the current and future organizations and sectors (De Vries et al., 2001; Fugate et al., 2004; Van der Heijde and Van der Heijden, 2006; Van Vuuren, 2012). Vanhercke et al. (2014) distinguished three approaches to employability: the perceived employability approach, the competence-based employability approach, and the dispositional employability approach. The perceived employability approach emphasizes the perception of one’s chances and opportunities in the labor market (Berntson et al., 2006; De Cuyper et al., 2011) and relates to the perceived ease to move, and having job alternatives. The competence-based approach is related to one’s skills and competencies to obtain and retain work or to create new work if necessary (Van der Heijde and Van der Heijden, 2006; Van der Heijden et al., 2018). The dispositional approach is merely focused on the innate abilities or characteristics of people that help them to find and keep a job, and to continue working in the future (Fugate and Kinicki, 2008). One could argue that age could also be a disposition but this is not what Fugate and Kinicki (2008) meant. The dispositional approach is more about dimensions like “work and career resilience,” and “work identity” and these may change over time and with age.

Forrier et al. (2015) considered employability as a chain of these different approaches that are conceptualized as stages in the employability process. More specifically, in their view, employability starts with the dispositional aspects (but these can also be considered as antecedents), it will be further developed in the competence-based aspects, and it results in the perceived chances and movement (mobility) in the labor market (Forrier et al., 2015), ultimately resulting in actual work and mobility (Forrier and Sels, 2003). These aspects can be measured among employees themselves (self-perceptions), but also among their supervisors, or different stakeholders (perceptions of others, such as one’s colleagues or relatives). However, in line with Fleuren et al. (2016), for our study we considered employability to be an individual-level construct and, in line with Veld et al. (2015), focused on self-perceived employability as our outcome variable.

Vitality, being the second component of sustainable employability, is a characteristic that refers to an individual’s levels of energy, vigor, and resilience at work (Bakker et al., 2002; Schaufeli et al., 2002a,b; Schaufeli and Bakker, 2003). It is often considered in the context of work engagement, in which vitality is one of the three components, also called “vigor,” in addition to the other components absorption and dedication (Schaufeli and Bakker, 2007). In the context of work-related flow (Bakker, 2001), vitality refers to happiness, and is differentiated from intrinsic motivation and absorption. According to Van Vuuren (2012), vitality relates to happiness, aliveness and intrinsic motivation at work. Ryan and Frederick (1997) already related it to well-being in an organismic manner (both mentally and physically). In all, vitality is considered to be an important element of sustainable employability (Semeijn et al., 2015), which is also confirmed in empirical studies (e.g., Brouwers et al., 2015; Van Scheppingen et al., 2015; Van Steenbergen et al., 2016; Semeijn et al., 2019).

Finally, work ability, being the third component of sustainable employability, refers to the degree to which individuals, given their health, are mentally, physically, and socially able to work now and in the near future (Ilmarinen et al., 2005). In other words, Ilmarinen (2006) argued that work ability builds on the balance between personal resources and work demands and that the concept is mainly related to people’s health and functional capacity. Although the three distinguished components of sustainable employability are correlated (see Van Vuuren et al., 2011), the components also have their own and independent value in the phenomenon of sustainable employability (Semeijn et al., 2015). In our study, we measured work ability by means of a single item for general health, which is a *proxy* for work ability. In doing so, we followed Martinez et al. (2009) who regarded health status as a proxy for work ability given its major role in determining work ability. Based on a cross-sectional analysis and from examining 12 month follow-up data of two large samples, Ebener and Hasselhorn (2019) confirmed that work ability was correlated substantially with general health.

### 1.3. Organizational learning climate

In line with the P-E fit perspective on sustainable employability, scholars such as De Lange et al. (2013) stated that a facilitating working context is essential for sustainable employability. This can also be understood from the Self-Determination Theory (SDT) (Ryan and Deci, 2000; Gagné and Vansteenkiste, 2013) which indicates that people are by nature motivated to unfold and develop themselves, but need a facilitating environment to do so (see also Van den Broeck et al., 2014). A working context that embraces, among other things, a climate that stimulates employees to learn, can thus enhance their (sustainable) employability (Van den Broeck et al., 2014). Such a climate is referred to as the learning climate of an organization (Bartram et al., 1993; Nikolova et al., 2014). Learning climate was referred to by Nixon (1991) as perceptions of work settings that may help or hinder learning at work. Previous empirical work has already indicated a positive relationship between learning climate and employability (Van der Heijde et al., 2018), between lifelong learning and sustainable employability (Van Vuuren et al., 2011), and between learning in the workplace and sustainable employability (Van der Heijden et al., 2016).

Bartram et al. (1993) developed the Learning Climate Questionnaire, consisting of seven dimensions. Van der Heijden et al. (2009), in their empirical research investigating key predictors for employability enhancement, used two of these dimensions of learning climate, namely: (lack of) time for learning, and perceived team support for learning (or team style). Perceived team support for learning refers to perceptions of opportunities to learn from expert colleagues. In the current study, in line with Van der Heijden et al. (2009), we also incorporated perceived team support, but instead of the (lack of) time for learning, we decided to include another one of the seven dimensions of Bartram et al.’s (1993) operationalization of learning climate, namely: the perceived given opportunities to develop which refers to the perceptions of opportunities to learn new jobs, and to do a variety of types of work at the workplace (Bartram et al., 1993; Cunningham and Iles, 2002; Van der Heijden et al., 2009, 2015). More specifically, we argue that lack of time for learning, being an aspect of learning climate, can also be the consequence of a high workload (e.g., Coventry et al., 2015), which implies that this could have affected our results unintentionally. We expect that the two dimensions of learning climate that are included for the current study can give the most relevant clues about a stimulating learning environment in the organization, in the light of sustainable employability. In particular, we argue that it depends on your team and on the opportunities you perceive within your team whether you experience the learning climate as positive or stimulating. Hence our first and second hypotheses were formulated as follows:

*Hypothesis 1:* The organizational learning climate dimension “team support for learning” is positively related to self-perceived employability (a), vitality (b), and work ability (c).

*Hypothesis 2:* The organizational learning climate dimension “perceived developmental opportunities” is positively related to self-perceived employability (a), vitality (b), and work ability (c).

### 1.4. Career commitment

Career commitment refers to “*one’s attitude toward one’s profession or vocation”* (Blau, 1985, p. 278) and indicates a motivational career resource (Halbesleben et al., 2014) that is beneficial in attaining career goals (Hirschi et al., 2018; Van der Heijden et al., 2021). Van der Heijden et al. (2021) argued that employees who portray individual agency (who are actively participating in their own life and career) are better able to protect and further enhance their sustainable employability. In the current study, based on the premises of the SDT (Ryan and Deci, 2000; Gagné and Vansteenkiste, 2013), we again argue that career commitment will be beneficial for the sustainability of an employee’s employability, as higher scores on career commitment, that represent a type of individual agency, will be related to higher scores on indicators of sustainable employability. In earlier studies, career commitment was already related to turnover intentions (Kim et al., 2016) and to employability, using a competence-based approach to its operationalization (Van der Heijden et al., 2022). Hence our third hypothesis was formulated as follows:

*Hypothesis 3:* Career commitment is positively related to self-perceived employability (a), vitality (b), and work ability (c).

In the light of the P-E fit perspective on sustainable employability that we adopted, we expect that career commitment (Person) also interacts with organizational learning climate (Environment) in their effect on sustainable employability. Career commitment is already known as a moderator in the relationship between person-job fit and innovative work behavior (e.g., Huang et al., 2019). In our study, we tested its interactive role in the relationship between learning climate and sustainable employability. More specifically, we hypothesized that individuals who actively participate in their own lives and careers (i.e., are high in career commitment) will benefit relatively more from a sound organizational learning climate compared to employees who are less committed to their career.

*Hypothesis 4:* Career commitment moderates the positive relationships between organizational learning climate and self-perceived employability (a), vitality (b), and work ability (c), such that these relationships are stronger when the employee is more committed.

### 1.5. Age

Regarding the possible role of age, a fair amount of research indicates that the aging process is usually accompanied by a decline in physical and mental capacities (Ilmarinen, 2006; Truxillo et al., 2015; Van der Mark-Reeuwijk et al., 2019; De Lange et al., 2020). However, the effect of age-related stereotyping seems to play a larger role in these results than the aging process itself (Lamont et al., 2015). In other words, several studies have already indicated that age as such has no detrimental influence, but that the work context often does not facilitate older employees equally compared to younger ones (Williams van Rooij, 2012; Lazazzara et al., 2013). For example, older employees are more likely to find themselves in jobs with little learning potential than their younger colleagues, often due to age-related stereotypes (Van der Heijden, 2005; Zinsmeister and Meerman, 2014). In line with Mulvey et al. (2010, p. 597), we consider age-related stereotyping as “the attribution of traits to individuals based on group membership,” in this case age group (cf. Toomey and Rudolph, 2017). This form of stereotyping can in principle entail positive or negative attributions, although age-related stereotyping tends to be mostly negative for *older* employees, leading to discrimination and exclusion (Mulvey et al., 2010). In this regard, it can lead to negative employee and applicant evaluations (Finkelstein and Burke, 1998), as well as to decreased ratings of self-efficacy, job satisfaction, performance, and to lower learning and development scores, or to increased retirement intentions (Weber et al., 2019). For our study, this means that we expect a negative age effect on two of the three components of sustainable employability, namely on self-perceived employability and work ability (measured in terms of health; see the section “2. Materials and methods”). Self-perceived employability is known to be susceptible to being affected by age-related stereotyping (De Lange et al., 2021), and health is inevitably (more or less) declining, in general, with age (Van Vuuren, 2012; Van Vuuren and Marcelissen, 2013). However, we do not expect an age effect on vitality, as people of all ages can be equally vital (e.g., Van Vuuren et al., 2011). Based on the outline above, we formulated the following hypothesis:

*Hypothesis 5*: Age relates negatively with self-perceived employability (a) and work ability (c).

Looking at the effect of age from our P-E fit perspective, we argue that age, similar to the moderating effect of career commitment, will be a strengthening factor in the relationship between organizational learning climate and sustainable employability. More specifically, in line with Bal et al. (2013), we build on a contingency perspective to justify our hypothesized moderation effect of age in this relationship. A contingency perspective on the impact of learning climate in organizations can be used to explain micro- or individual-level outcomes (cf. Delery and Doty, 1996), such as self-perceived employability and work ability. In other words, we posit that the impact of organizational learning climate factors is not only dependent on macro-level factors, such as organizational strategy, but also on micro-level factors, such as the employees’ needs, abilities and preferences across the life-span, and thus upon their age.

Life-span theorizing (Baltes, 1987) states that aging usually involves a relative loss of individual resources (e.g., Takeda et al., 1992; Faulkner et al., 2007; Salthouse, 2013), which might endanger the sustainable employability of older employees. Therefore, we contend that, in terms of optimal resource allocation (Baltes and Lang, 1997), a strong organizational learning climate is particularly important for older employees. Specifically, building on selective optimization with compensation (SOC) theory (Baltes and Baltes, 1990; Ebner et al., 2006; De Lange et al., 2011), we argue that the availability of a strong organizational learning climate, that enhances the employee’s ability to adopt and to fine-tune specific strategies for minimizing losses and maximizing gains, is particularly beneficial for older employees. When compensating for losses, older employees may shift their preference from extrinsically rewarding job features (such as competition with peers and promotions) to more intrinsically rewarding job features (such as opportunities to develop) in order to enhance their career sustainability (De Vos et al., 2020; Van der Heijden et al., 2020).

Indeed, previous research has indicated that employees who experience losses in their capabilities appear to use SOC strategies in order to protect their subjective career success (Wiese et al., 2002; Guillén and Kunze, 2019). Since we expect this phenomenon of adopting SOC strategies only to occur when older employees experience losses, we expect this to happen only in terms of self-perceived employability and work ability as both are expected to decline with age. Moreover, in order to successfully employ a desired SOC strategy, it is also necessary that the organization supports these individual strategies (Bal et al., 2013). Therefore, by adopting a P-E fit perspective (Kristof, 1996; Schneider, 2001; Kristof-Brown and Billsberry, 2013) and integrating this with life-span theorizing (Baltes, 1987), as well as building on the notion of sustainable careers (De Vos et al., 2020; Van der Heijden et al., 2020), we formulated the following hypothesis:

*Hypothesis 6:* Age moderates the positive relationship between organizational learning climate, on the one hand, and self-perceived employability (a) and work ability (c), on the other hand, such that these relationships are stronger when the employee is older.

In addition, based on and continuing this line of reasoning, we also hypothesized a three-way interaction effect. In particular, we expect that the positive impact of the organizational learning climate on sustainable employability is stronger for people who have a higher career commitment and who are older at the same time (see also Van Dam et al., 2017; De Lange et al., 2020).

Particularly, and in line with above, by adopting a P-E fit perspective (Kristof, 1996; Schneider, 2001; Kristof-Brown and Billsberry, 2013) as the overarching framework in our study, and building on the notion of the sustainable career paradigm, we argue that employees who are more committed to their career (i.e., active agents in their own career course) and who are older at the same time are more focused on protecting and further enhancing their sustainable employability (De Vos et al., 2020; Van der Heijden et al., 2020).

First, borrowing from the assumptions underlying conservation of resources (COR) theory (Hobfoll, 1989), we contend that employees who have a higher degree of career commitment and who are older are more likely to benefit from a stronger organizational learning climate. According to the *principle of resource investment*, a contextual resource (in our case organizational learning climate) that can help an individual to attain a certain career goal or satisfy particular needs (in our case employability and work ability) is especially valuable for them. However, simultaneously, following the *principle of the primacy of resource loss*, the employees will be more likely to be able to protect and/or ideally further enhance their sustainable employability if they manage to adapt well to losses. Therefore, we argue that individuals who are strongly committed to their career (personal resource) are more likely to value and utilize the contextual resource of the organizational learning climate as it helps them to enhance their level of sustainable employability.

Second, building on self-determination theory (SDT) (Ryan and Deci, 2000; Gagné and Vansteenkiste, 2013), we argue that individuals are inclined to act in order to master both internal and external forces (i.e., strive for autonomy). Second, they tend to strive for growth, development, and integrated functioning (i.e., strive for competence). Third, individuals need a supportive environment (relatedness) (Deci and Ryan, 2000) in order to realize person–career fit (Parasuraman et al., 2000), which may be reflected in terms of a high level of sustainable employability (De Vos et al., 2020; Van der Heijden et al., 2020).

In summary, in line with COR theory (Hobfoll, 1989) and SDT (Ryan and Deci, 2000; Gagné and Vansteenkiste, 2013), we expect that older employees with a high degree of career commitment are more inclined to invest time in getting the most out of a strong organizational learning climate (Hobfoll, 2001), thereby creating so-called resource caravans (e.g., Westman et al., 2004) that may result in a higher level of self-perceived employability and work ability. Therefore, we formulated the following hypothesis:

*Hypothesis 7:* A three-way interaction between organizational learning climate, career commitment and age will be related to self-perceived employability (a) and work ability (c). Specifically, the positive relationship between organizational learning climate, on the one hand, and self-perceived employability (a) and work ability (c), on the other hand, will be stronger for people who are both more committed to their careers and older.

**FIGURE 1:**
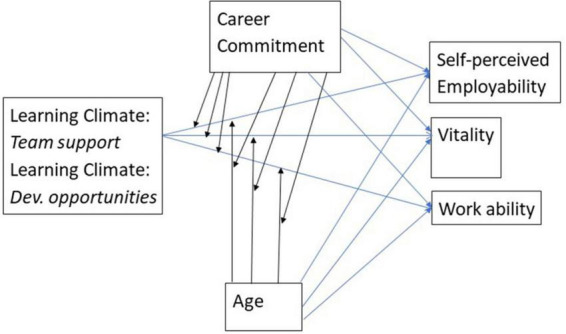
Conceptual model.

Figure 1 represents the seven hypotheses in a conceptual model. In this model that forms the basis of our empirical work, one can see that first, organizational learning climate [which was operationalized by means of two factors (i.e., team support for learning and developmental opportunities)], career commitment, and employee age were hypothesized as antecedents of three indicators of sustainable employability (i.e., workers’ self-perceived employability, vitality and work ability). Second, two-way interaction effects between career commitment and age on the one hand, and each of the two organizational learning climate factors were taken into account. Third, three-way interaction effects between each of the two organizational learning climate factors, age, and career commitment, on the one hand, and all three indicators of sustainable employability, on the other hand, were hypothesized.

## 2. Materials and methods

### 2.1. Sample and data collection

A total of 211 support staff employees from a Dutch university completed an online survey (50.0% response rate; Van der Heijden et al., 2006). To reduce possible common-method bias, the anonymity of the participants was ensured and respondents were invited to be as honest as possible in their answers (Podsakoff et al., 2003). The final sample included 108 (51,0%) men and 103 (49,0%) women. The mean age of the participants was 46 years of age (SD = 8; ranging from 24 to 62). Of the participants, 77.3% were older than 40 years. Moreover, 32.7% of participants were over 50 years old. Three quarters of the respondents had completed higher vocational education or university education. The mean labor market experience of the participants was 14.88 years (SD = 2.66). Finally, 57.8% of the respondents were working full-time (see Supplementary Appendix Table 1 for the mean and standard deviation of Age, Gender, and Educational Background).

All survey scales were previously used and thoroughly validated in a large international research project concerning employability in seven countries in Europe (including the Netherlands; Van der Heijden, 2005).

### 2.2. Method of measurement and scales

The questionnaire started with a number of questions assessing the participant’s age (calendar age), gender (one for men and two for women), and educational background (measured by the highest level of education attained (1 for primary education to 6 for university level).

#### 2.2.1. Self-perceived employability

Self-perceived employability was assessed by means of the individual’s self-reported labor market position. It was rated on a five-point Likert scale ranging from: (1) “very difficult” to (5) “very easy.” Two items were used to measure perceived labor market position of the individual employee [based on Lindstroöm (1997)], that were quite strongly correlated (*r* = 0.714, *p* < 0.001), including: “How easy would it be for you to find a suitable job with another employer?” and “How easy would it be for you to find a job as good as the one you have now with another employer?.”

#### 2.2.2. Vitality

Vitality was measured with the work enjoyment subscale of the work-related flow scale (Bakker, 2001). The subscale includes four items for work enjoyment (Cronbach’s α = 0.90), for example: “When I am working very intensely, I feel happy.” It was rated on a seven-point Likert scale ranging from: (1) “never” to (7) “always.”

#### 2.2.3. Work ability

Work ability was assessed by means of a single item, indicating general Health, based on the SF-36-Health scale (Ware and Sherbourne, 1992). The item was formulated as follows: How do you consider your health in general? It was rated on a five-point Likert scale ranging from: (1) “bad” to (5) “excellent.” Although single-item measures are often considered less preferable, they also have advantages, for example for being parsimonious in data gathering and making a construct easy and less threatening to rate (DeSalvo et al., 2006). Health as a proxy for work ability has been included in previous studies with single-item measures as well (Oakman et al., 2018) and has proven to be a reasonable alternative to the WAI (see e.g., Jääskeläinen et al., 2016).

#### 2.2.4. Organizational learning climate

Organizational learning climate, as perceived by the individual employee, was operationalized by means of two dimensions, namely: team style or perceived team support for learning Cronbach’s α = 0.84) and perceived opportunities for development (Cronbach’s α = 0.78) (Bartram et al., 1993). The items were scored on a five-point Likert scale ranging from: (1) “never applicable” to (5) “always applicable.” Sample items were: “If I have a question about my job, there is someone available to ask” (perceived team support). And “There are lots of different ways to learn new jobs here” (opportunities for development).

#### 2.2.5. Career commitment

Career Commitment was measured by Van der Heijden (2005) four-item scale. It is based on Jaskolka et al.’s (1985) measurement of job involvement. Items were scored on a five-point scale ranging from: (1) “totally disagree” to (5) “totally agree.” The scale, for example, includes the following item: “The greatest satisfaction in my life comes from achieving my professional/career objectives.” (Cronbach’s α = 0.75).

#### 2.2.6. Age

Age was measured by means of calendar age.

### 2.3. Method of analysis

SPSS for Windows version 28.0 was used to analyze the data. Standard descriptive statistics were computed to describe the study’s variables (see Supplementary Appendix Table 1). Supplementary Appendix Table 1 also shows all intercorrelations between the variables used in our study. Subsequently, hierarchical regression analyses were conducted for the three aspects of sustainable employability. All three analyses examined the relationships between the personal background variables, i.e., age, gender, and educational background (Step 1), the organizational learning climate and career commitment variables (Step 2), the two-way-interaction effects (Step 3), and the three-way-interaction effects (Step 4), on the one hand, and the three components of sustainable employability, on the other hand. For Step 3 and 4, we first calculated the interaction variables to include in our analysis. We used the mean centered values of the original variables. To measure the effects of the two-way interactions we calculated these two-way interactions of (1) Age with Team Support, (2) Age with Developmental Opportunities, (3) Age with Career Commitment, and (4) Career Commitment with Team Support as well as (5) Career Commitment with Developmental Opportunities. We included these interactive variables in Step 3 of the models in Supplementary Appendix Table 2. To measure the effects of the three-way interactions, we calculated these based on the mean centered values of (1) Age with Career Commitment and Team Support, and (2) Age with Career Commitment and Developmental Opportunities. We added these resulting three-way interaction variables to test their effects in Step 4 of the models in Supplementary Appendix Table 2.

## 3. Results

### 3.1. Descriptive analyses

Supplementary Appendix Table 1 shows the descriptive statistics and correlations between the variables of our study. In line with our expectations, the three components of sustainable employability are significantly correlated, but not enough (not more than 30%) to justify a distinction between them. Educational background has a generally unexpected negative association with self-perceived employability. Moreover, higher educated employees and females report higher levels of work ability in comparison with lower educated employees and males.

With regard to the predictors in our research model, that is organizational learning climate and career commitment, we noticed that the two distinct learning climate dimensions have positive associations with all three aspects of sustainable employability, while career commitment is only associated with vitality. Age is negatively associated with self-perceived employability and work ability, but not with vitality. In addition, age is negatively associated with both our learning climate dimensions, but not with career commitment.

### 3.2. Hierarchical regression analyses

Supplementary Appendix Table 2 shows the results of the hierarchical multivariate regression analyses for all three dependent variables in our model.

**FIGURE 2:**
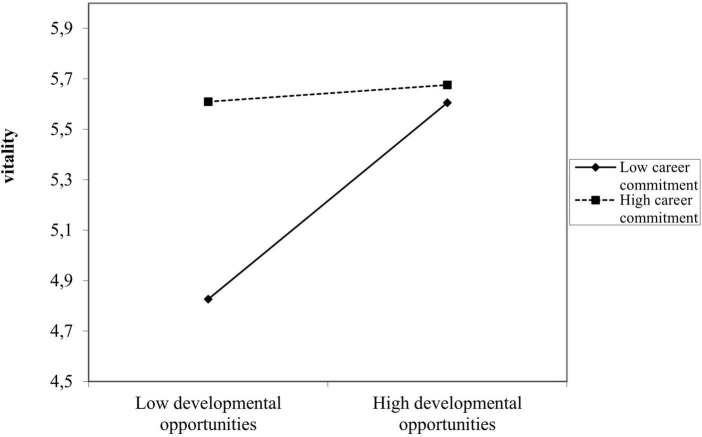
Two-way interactive effect of developmental opportunities with career commitment for vitality.

No direct effects of the learning climate dimension “team support for learning” were found, which means **we cannot confirm Hypothesis 1 with our data**. For “perceived developmental opportunities,” direct effects are visible for all aspects of sustainable employability, especially in the Step 4 models (with β = 0.158; *p* < 0.05 for self-perceived employability, β = 0.213; *p* < 0.01 for vitality, and β = 0.237; *p* < 0.01 for work ability), which indicates that our **Hypothesis 2 is fully confirmed with our data.** Subsequently, our results indicate that our expectation in **Hypothesis 3 is only partially confirmed**: career commitment is positively related to vitality (β = 0.227; *p* < 0.01), but not to self-perceived employability, nor to work ability. However, **Hypothesis 4 is not confirmed**; no positive two-way interactions were found between the perceived developmental opportunities and career commitment in predicting components of sustainable employability. However, a negative two-way interaction effect for vitality appeared. In Figure 2, we visualize this negative effect between perceived developmental opportunities with career commitment in the light of vitality.

As Figure 2 shows, a low score on perceived developmental opportunities can be compensated for by means of a high score on career commitment when looking at the effect on vitality. Moreover, a high score on perceived developmental opportunities is especially beneficial for staff who score low on career commitment. For employees who are highly committed to their career, the degree of developmental opportunities is not important for their vitality. These employees appear to be vital regardless of the extent to which they experience developmental opportunities.

**Hypothesis 5 is fully confirmed with our data**; age is negatively related to self-perceived employability and work ability in all regression models. Supplementary Appendix Table 2, however, also indicates that **Hypothesis 6 is not confirmed,** which deals with the moderating effect of age in the relationship between learning climate and the three components of sustainable employability. More specifically, no interaction effects were found for any of the sustainable employability outcomes.

**FIGURE 3:**
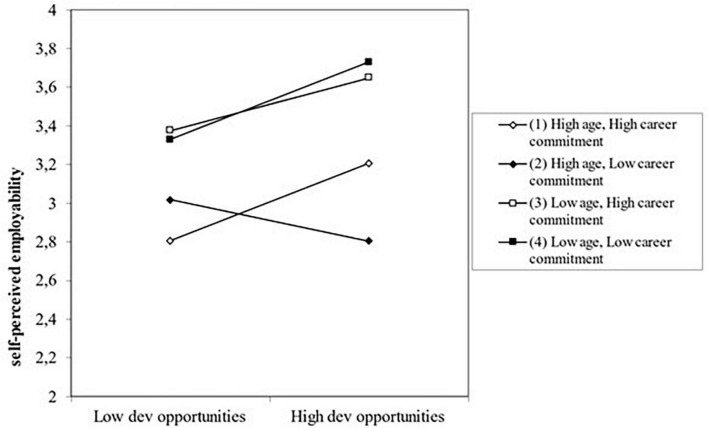
Three-way interactive effect of developmental opportunities with career commitment and with age for self-perceived employability.

Finally, **Hypothesis 7 is partially confirmed with our data**. In particular, the positive relationship between the organizational learning climate dimension “perceived developmental opportunities” and self-perceived employability appeared to be stronger for people who are more committed to their career as well as older (β = 0.157; *p* < 0.05). We could not find such an effect when work ability was the outcome variable. Nor did we find this positive relationship between the organizational learning climate dimension “team support for learning” on the one hand and self-perceived employability and work ability on the other hand, when people are both more committed to their career and older. We visualize this three-way interaction effect of developmental opportunities x career commitment x age for self-perceived employability in Figure 3.

As Figure 3 shows, older employees perceive *less* employability than younger employees, especially when they do *not* experience many developmental opportunities and at the same time are highly committed to their careers, or when they experience *many* developmental opportunities and are not so much committed to their careers at the same time. For the younger employees, the degree to which they are committed to their career does not seem to matter. Only the level of perceived developmental opportunities appears to be associated with their self-perceived employability. In other words, for these young workers, whether they are highly committed to their career or not, a higher level of perceived developmental opportunities is related to their self-perceived employability.

## 4. Discussion

### 4.1. Reflection on outcomes

The results of our study show that, in general, organizational learning climate, career commitment, and age are related to sustainable employability outcomes. That is, employees’ levels of self-perceived employability, vitality, and work ability increase when the work context facilitates learning, especially as predicted by Hypothesis 2 by providing employees with more development opportunities. Unexpectedly, no such effect could be found as predicted by Hypothesis 1 for the dimension of team support for learning. Our findings regarding the importance of developmental opportunities are in line with earlier scholarly research by Van der Heijden et al. (2015), and Van der Heijden et al. (2018). It is also in line with the work by Bal et al. (2013) and Van Dam et al. (2017), because it underlines the benefits of a facilitating work context. Moreover, it suggests that the provision of developmental opportunities is beneficial to all employees, regardless of their age, gender, or educational background. The results of our study also partially support Hypothesis 3 that employees who are highly committed to their careers are better at sustaining their employability, at least if vitality is taken as the outcome measure. This outcome contrasts with the work of Hirschi et al. (2018) who showed that career involvement was important for career success, operationalized in terms of salary and career satisfaction in several meta-analyses. However, in line with our findings, Van der Heijden et al. (2021) also found no main effect of career commitment on subjective career success.

Moreover, similar to our scholarly work, Van der Heijden et al. (2021) found that age differences played a significant role in the relationships between career commitment and career success outcomes. Our results also partly confirm Hypothesis 5 and indicate that age has an effect on the relationships between career commitment and developmental opportunities on the one hand and self-perceived employability on the other. Particularly for younger employees, we found that the relationship between developmental opportunities and self-perceived employability hardly changed as a result of the level of career commitment, but it did for older employees. Specifically, the more committed they are to their career, the stronger the relationship between developmental opportunities and self-perceived employability. Yet their self-perceived employability scored lower than that of younger employees. Age was also important for one’s work ability. As in other studies, we found that the older the employees, the lower they estimate their work ability and employability (Hypothesis 4) (Van Vuuren, 2012; Van Vuuren and Marcelissen, 2013; De Lange et al., 2021).

According to the P-E fit perspective (Kristof, 1996; Schneider, 2001; Kristof-Brown and Billsberry, 2013) and the sustainable career paradigm (Van der Heijden, 2005; De Vos et al., 2020), employees who are active agents in their own career course are better able to use their work environment to enhance their sustainable employability. Based on these theoretical frameworks, we expected that employees who are more committed to their careers would benefit more from a strong learning climate for their sustainable employability, but we found no support for this assumption. Rather we found that for employees who are highly committed to their careers, the level of developmental opportunities made no difference to their vitality. They appeared to be vital regardless of the extent to which they experienced developmental opportunities. However, for the employees who were less committed to their careers, the level of developmental opportunities they experienced counted. The more developmental opportunities they perceived, the more vital they were.

The latter outcome contradicts the proposition of Van der Klink et al. (2016) who stated that employees’ attitude and motivation need to exploit the opportunities that an organization offers them. Our results showed that an employee’s attitude and motivation–if high–can be sufficient to be vital, but if employees are not motivated enough, it is important as an organization to offer developmental opportunities in order to protect their vitality. Furthermore, our results confirmed the P-E fit perspective on sustainable employability that states that both the individual (Person) and organization (Environment) are necessary, but only for older employees. For the latter, we found a three-way interaction effect between developmental opportunities, career commitment and age, in light of their self-perceived employability. In general, we found significant positive effects of both organizational learning climate and career commitment on sustainable employability. The same applies to the impact of age: age appeared to have a direct negative effect on self-perceived employability and work ability. With these outcomes, our study specifies the understanding of the P-E fit perspective for sustainable employability: employees’ attitude and motivation and their perception of the developmental opportunities that an organization offers to them are both, but separately, important for their sustainable employability. Moreover, only when the employees are older or less committed to their careers does the interaction with (developmental) opportunities have an additional effect.

Our findings leave room for exploring other factors that may explain aspects of sustainable employability. This can include different person-related resources, such as hope, resilience, optimism and self-efficacy (also known as “Psycap”) (Luthans et al., 2007), or different environmental factors, such as the actual HR practices in the organization (Semeijn et al., 2015). It is possible that these factors can explain more variance and/or play a more important role in research adopting a P-E fit perspective. For example, Veld et al. (2015) explored the *value of actual HR practices* aimed at development and mobility together with the *willingness* for development and mobility of the employees. Their models accounted for 22% of explained variance.

### 4.2. Practical implications

The current study demonstrates that age is negatively related to self-perceived employability and work ability, but not related to vitality, which is in line with previous studies (e.g., Van Vuuren et al., 2011; Semeijn et al., 2019). When it comes to the provision of a strong organizational learning climate, employers, and on their behalf HR professionals, should not differentiate between employees based on their age, gender or educational background. Our outcomes show that an organizational climate that facilitates learning (indicated by perceived developmental opportunities) can enhance sustainable employability of all categories of employees. Therefore, organizations need to create a strong organizational learning climate by implementing developmental practices that can fulfil the learning needs of *all* employees (however, tailor-made|) (e.g., Van Vuuren et al., 2015). Moreover, a higher level of career commitment also enhances the sustainable employability of employees. This indicates that employees themselves also need to invest in their careers in order to protect and ideally further enhance their sustainable employability. The current study also emphasizes the importance of the fit between person and environment (incorporated in our study through the interaction between developmental opportunities and career commitment) between the organization and the individual employee which Van der Klink et al. (2016) emphasized in their definition of sustainable employability, and what has already been confirmed in previous empirical studies (e.g., Veld et al., 2015). For management practice, one could therefore think of explicitly discussing employees’ current career plans, needs and ambitions, on a regular basis, in order to stay aligned and to make the P-E fit more explicit, possibly and if relevant also to integrate it in the formal meetings between employee and supervisor, in which results and development are discussed.

### 4.3. Study limitations and recommendations for future research

Based on our results, we recommend to further examine the P-E fit perspective on sustainable employability, building on Clarke and Patrickson (2008). Our findings indicate the relevance of interactions between environmental and individual factors in light of employees’ sustainable employability outcomes. It is therefore important to further explore the value of these and other potentially relevant interactions, such as mentioned in the previous section, in future research. However, we need to take into account the case study character of this scholarly work; our sample consists of the support staff of a particular university in the Netherlands. We therefore need to draw a cautionary note on the generalizability of our findings to the general population. Collecting the data within one organization contributes to the internal validity of our study, yet it comes at the expense of its external validity. For instance, the (higher educated part of the) participants in our study perceived relatively less labor market opportunities in contrary to the (higher educated part of the) support staff at other Dutch universities (Van Vuuren et al., 2013). Considering this, further research incorporating other working populations is needed before we can more safely conclude about the generalizability of our findings to other occupational settings and to other countries (Fouad and Arbona, 1994).

Another limitation comprises the cross-sectional design used in our study, which only collected survey data. This opens the possibility of response set consistencies. In order to assess both cross-sectional validity and causality, the model should be investigated in other contexts as well as longitudinally. Future work with multi-wave designs is needed to better elaborate on the stability and change of the variables, and on possible cross-lagged relationships than the current cross-sectional approach (De Lange et al., 2005; Semeijn et al., 2019). Over time, this will allow for more insights into the interactionist process.

In addition, all variables were measured using self-reports, which may have led to an increase in the correlations between the measured constructs, due to so-called “common method bias,” which made the relationships found stronger (Podsakoff et al., 2003). However, this risk has been mitigated by ensuring the anonymity of the respondents and giving them the option to stop filling out the survey at any time. Nevertheless, future studies could consider combining the use of (self) reports, for example on learning climate as well as perceived employability. This could increase our understanding of the discrepancies between rater groups, which may also shed more light on possible (perceived age-related) stereotyping in combination with measures of stereotyping *per se* (cf. Van Selm and Van der Heijden, 2013). Consideration could also be given to including more objective measures, such as for work ability, but also for learning climate in which the developmental possibilities could be assessed more objectively in terms of their presence within an organization, and making use of various sources.

In addition, we also need to take into account that we measured work ability with a proxy (self-perceived health), which has some disadvantages when it comes to understanding its value. For example, health can also be considered to be an antecedent of sustainable employability, especially for work ability (Van den Berg, 2010). In a similar vein, with regard to the operationalization of the organizational learning climate, as we included only two elements of the seven dimensions of the Learning Climate Questionnaire (Bartram et al., 1993), namely development opportunities and team support for learning. More research is needed to include broader operationalization of this construct. Although Mikkelsen and Grønhaug (1999) pointed out that the seven dimensions may overlap, each of them also captured something unique, according to these scholars. Further research is needed to investigate whether other dimensions of organizational learning climate are also important in light of sustainable employability. A recent Dutch study of Van der Weide et al. (2022) about learning culture in Small- and Medium-sized Enterprises (SMEs) distinguished six building blocks that they believe are essential for the development of a learning culture within SMEs: (1) job content, (2) time, facilities, and (psychological) safety, (3) collaboration and team development, (4) leadership, (5) organizational design, and (6) connection to the external environment. Finally, the findings of the current study may have been affected by the healthy employee bias (McMichael et al., 1986). Therefore, the inevitable exclusion of unhealthy employees during data collection may have influenced the results of the current study in terms of relationships being stronger or more positive as compared to the whole population. Therefore, future research is needed to make more accurate estimations of the possible healthy employee bias at hand.

## 5. Conclusion

Our findings confirm the relevance of adopting a P-E fit perspective on sustainable employability, and of considering the possible role of age in the relationships under study. We found that both individual (Person) and work-related (Environment) factors, and under certain circumstances in interaction with one another, influence the employees’ sustainable employability. In particular, our study shows that employees’ commitment, and the learning climate that an organization offers to them are both important for the employees’ career potential. Moreover, only when the employees are older or less committed to their careers does the interaction between age and career commitment with organizational learning climate have an additional effect.

The findings of the current study provide us with more insight into how employers can use contextual factors, such as creating a strong organizational learning climate, to increase the sustainable employability of their employees, regardless of their age, gender, or educational background. In addition, our findings also show how important it is for employees to feel committed to their careers. Especially considering the benefits for their vitality, and in case they are older, particularly in combination with the amount of perceived developmental possibilities, also for their self-perceived employability. Finally, employers and HR professionals need to keep in mind that, even although age is negatively related to self-perceived employability and work ability of employees, their vitality remains just as good as for their younger counterparts. Therefore, age does not have to play a negative role in terms of the opportunities to protect and further enhance the sustainable employability of employees.

## Data availability statement

The raw data supporting the conclusions of this article will be made available by the authors, without undue reservation.

## Ethics statement

Ethical review and approval was not required for the study on human participants in accordance with the local legislation and institutional requirements. The patients/participants provided their written informed consent to participate in this study.

## Author contributions

BV, TV, and JS developed the study design. BV was responsible for the coordination of the study project and its data collection. JS prepared the data analysis with support from TV and BV. TV and BV wrote the first draft of the manuscript. All authors shaped the later drafts of the manuscript, read, and approved the submitted version.
