# Supplementary material for: With a little help from my friends: adopting a P-E fit perspective in understanding the value of organizational learning climate for sustainable employability
Source: Front Psychol. 2023 Apr 17;14:1128535. doi: 10.3389/fpsyg.2023.1128535 (PMC10150123; doi:10.3389/fpsyg.2023.1128535)
Supplement: Supplementary file 1 [file Data_Sheet_1.docx]

APPENDIX 1

Table 1. Descriptives and intercorrelations of all variables in our study

|  |  |  |  |  |  |  |  |  |  |  |
| --- | --- | --- | --- | --- | --- | --- | --- | --- | --- | --- |
| variables (N=350) | M | sd | 1 | 2 | 3 | 4 | 5 | 6 | 7 | 8 |
| (1) Age | 46.27 | 8.28 | 1.00 |  |  |  |  |  |  |  |
| (2) Gender (1-2) | 1.49 | 0.50 | -0.163* | 1.00 |  |  |  |  |  |  |
| (3) Educational Background (1-6) | 5.21 | 1.03 | 0.035 | -0.282** | 1.00 |  |  |  |  |  |
| (4) Learning Climate –  Team Support (1-5) | 3.37 | 0.56 | -0.201** | 0.155* | -0.084 | 1.00 |  |  |  |  |
| (5) Learning Climate – Developmental Opportunities (1-5) | 3.15 | 0.55 | -0.116 | -0.072 | 0.094 | 0.555** | 1.00 |  |  |  |
| (6) Career Commitment (1-5) | 2.45 | 0.67 | -0.083 | 0.030 | -0.049 | -0.079 | 0.020 | 1.00 |  |  |
| (7) Self-perceived Employability (1-5) | 2.66 | 0.70 | -0.390** | 0.055 | -0.134 | 0.124 | 0.156* | 0.061 | 1.00 |  |
| (8) Vitality (Happiness) (1-7) | 5.03 | 0.92 | 0.048 | 0.068 | -0.117 | 0.077 | 0.191** | 0.258** | 0.116 | 1.00 |
| (9) Work ability (Health) (1-5) | 3.23 | 0.77 | -0.316** | 0.159* | 0.016 | 0.196** | 0.263** | 0.104 | 0.304** | 0.235** |

* Correlations are significant at *p* < .05. ** Correlations are significant at *p* <.01
` Gender; Male = 1, Female = 2

APPENDIX 2

Table 2. Regression analysis: predictors of sustainable employability

|  |  | Self-perceived Employability | |  |  | Vitality (Happiness) | |  |  | Work ability (Health) | |  |  |  |  |  |  |  |  |  |
| --- | --- | --- | --- | --- | --- | --- | --- | --- | --- | --- | --- | --- | --- | --- | --- | --- | --- | --- | --- | --- |
| N=211 | step 1 | step 2 | step 3 | step 4 | step 1 | step 2 | step 3 | step 4 | step 1 | step 2 | step 3 | step4 |  |  |  |  |  |  |  |  |
| Control Variables |  |  |  |  |  |  |  |  |  |  |  |  |  |  |  |  |  |  |  |  |
| Age | -0.393** | -0.397** | -0.382** | -0.414** | 0.060 | 0.105 | 0.081 | 0.089 | -0.297** | -0.260** | -0.252** | -0.260** |  |  |  |  |  |  |  |  |
| Gender | -0.047 | -0.032 | -0.037 | -0.047 | 0.048 | 0.066 | 0.048 | 0.051 | 0.128 | 0.145* | 0.159* | 0.157* |  |  |  |  |  |  |  |  |
| Educational background | -0.134* | -0.146* | -0.143* | -0.136* | -0.106 | -0.116 | -0.118 | -0.121 | 0.062 | 0.046 | 0.046 | 0.046 |  |  |  |  |  |  |  |  |
| Independent Variables |  |  |  |  |  |  |  |  |  |  |  |  |  |  |  |  |  |  |  |  |
| Learning Climate -  Team Support |  | -0.039 | -0.033 | -0.059 |  | -0.029 | -0.050 | -0.041 |  | -0.001 | -0.020 | -0.024 |  |  |  |  |  |  |  |  |
| Learning Climate - Developmental Opportunities |  | 0.145 | 0.147 | 0.158* |  | 0.230** | 0.219** | 0.213** |  | 0.238** | 0.236** | 0.237** |  |  |  |  |  |  |  |  |
| Career Commitment |  | 0.019 | 0.034 | 0.029 |  | 0.253** | 0.221** | 0.227** |  | 0.075 | 0.064 | 0.066 |  |  |  |  |  |  |  |  |
| 2-way Interaction |  |  |  |  |  |  |  |  |  |  |  |  |  |  |  |  |  |  |  |  |
| AgexTeam |  |  | 0.031 | 0.053 |  |  | 0.085 | 0.078 |  |  | -0.019 | -0.014 |  |  |  |  |  |  |  |  |
| AgexDev |  |  | -0.073 | -0.087 |  |  | 0.005 | 0.014 |  |  | 0.106 | 0.106 |  |  |  |  |  |  |  |  |
| AgexCC |  |  | 0.053 | 0.049 |  |  | 0.102 | 0.098 |  |  | -0.088 | -0.093 |  |  |  |  |  |  |  |  |
| CCxTeam |  |  | -0.008 | -0.023 |  |  | 0.071 | 0.076 |  |  | -0.145 | -0.148 |  |  |  |  |  |  |  |  |
| CCxDev |  |  | 0.078 | 0.100 |  |  | -0.205** | -0.209* |  |  | 0.052 | 0.058 |  |  |  |  |  |  |  |  |
| 3-Way interaction |  |  |  |  |  |  |  |  |  |  |  |  |  |  |  |  |  |  |  |  |
| AgexCCxTeam |  |  |  | -0.142 |  |  |  | 0.060 |  |  |  | -0.016 |  |  |  |  |  |  |  |  |
| AgexCCxDev |  |  |  | 0.157* |  |  |  | -0.037 |  |  |  | 0.038 |  |  |  |  |  |  |  |  |
| Adj.*R²* | 0.156 | 0.161 | 0.150 | 0.162 | 0.004 | 0.104 | 0.131 | 0.125 | 0.102 | 0.152 | 0.155 | 0.148 |  |  |  |  |  |  |  |  |
| *F* (sign) | 13.979** | 7.702** | 4.363** | 4.126** | 1.308 | 5.083** | 3.886** | 3.310** | 8.979** | 7.263** | 4.511** | 3.800** |  |  |  |  |  |  |  |  |

* significant at *p* <.05. ** significant at *p* <.01
` Gender; *Male* =1, Female = 2
